# Plant Response to Engineered Metal Oxide Nanoparticles

**DOI:** 10.1186/s11671-017-1861-y

**Published:** 2017-02-06

**Authors:** Khwaja Salahuddin Siddiqi, Azamal Husen

**Affiliations:** 1 Department of Chemistry, Aligarh Muslim University, Aligarh, 202002 Uttar Pradesh India; 2 Department of Biology, College of Natural and Computational Sciences, University of Gondar, PO Box #196, Gondar, Ethiopia

**Keywords:** Metal oxide nanoparticles, Growth response, Antioxidant enzymes, ROS, Phytotoxicity

## Abstract

All metal oxide nanoparticles influence the growth and development of plants. They generally enhance or reduce seed germination, shoot/root growth, biomass production and physiological and biochemical activities. Some plant species have not shown any physiological change, although significant variations in antioxidant enzyme activity and upregulation of heat shock protein have been observed. Plants have evolved antioxidant defence mechanism which involves enzymatic as well as non-enzymatic components to prevent oxidative damage and enhance plant resistance to metal oxide toxicity. The exact mechanism of plant defence against the toxicity of nanomaterials has not been fully explored. The absorption and translocation of metal oxide nanoparticles in different parts of the plant depend on their bioavailability, concentration, solubility and exposure time. Further, these nanoparticles may reach other organisms, animals and humans through food chain which may alter the entire biodiversity. This review attempts to summarize the plant response to a number of metal oxide nanoparticles and their translocation/distribution in root/shoot. The toxicity of metal oxide nanoparticles has also been considered to see if they affect the production of seeds, fruits and the plant biomass as a whole.

## Review

### Introduction

Because of huge production and inadvertent use of nanomaterials, the whole environment is affected. Although, many of them are useful, some are toxic to plants, algae and microorganisms. They may, therefore, pose potential risk to the environment. Nanomaterials are frequently used in plant growth, cosmetics, drug delivery, photonic crystals, analysis, food, coatings, paints, bioremediation, catalysis and material science [1–12]. Keller and Lazareva [13] have reported that about 3000 tons of titanium dioxide (TiO_2_) nanoparticles are produced every year and more than 50% of which is used in personal care products [14]. Copper oxide (CuO) nanoparticles cause membrane damage to *Escherichia coli* as demonstrated by K^+^ leakage [15]. A study on Zebrafish (*Danio rerio*) has shown that as exposure days of TiO_2_ nanoparticles were increased, the number of viable embryos was decreased [16]. Some nanomaterials are toxic to flora and fauna as they are used to inhibit their growth to prevent further multiplication [17, 18]. Biouptake and accumulation of nanomaterials in plants may increase shoot length and decrease root length and their proliferation [19, 21]. The toxicity response depends on the concentration, particle size and shape of the nanomaterials [22]. Some studies have demonstrated that nanoparticle exposure improves free-radical scavenging potential and antioxidant enzymatic activities and alters microRNAs expression that regulates different morphological, physiological and metabolic processes in plants [22]. The toxicity of the free metal ions has been shown to be greater than that of the nanoparticles. For instance, silver nanoparticles are less toxic to plants than the silver ions [23] mainly due to greater solubility of AgNO_3_ and greater mobility of Ag^+^ ions in aqueous medium. Lipid peroxidation is an important parameter which indicates the cell membrane integrity [24–26]. Reactive oxygen species (ROS) generation is known to damage cell membrane through lipid peroxidation leading to ion leakage and disruption of the cellular metabolism leading to cell death. ROS also cause oxidative damage to photosynthetic apparatuses and biomolecules [27, 28]. Thus, plants protect cellular and sub-cellular system from the cytotoxic effects of active oxygen radicals with antioxidative enzymes (superoxide dismutase, SOD; catalase, CAT; peroxidase, POD and ascorbate peroxidase; etc.) and low molecular weight antioxidants (ascorbate, glutathione, proline, carotenoids, a-tocopherols and phenolics, etc.) and non-enzymatic components (carotenoids, ascorbate and tocopherol, etc.) [26, 29–31]. These components minimize the oxidative damage during exposure to metal oxide nanoparticles [18, 32, 33]. *Zea mays* exposed to CeO_2_ nanoparticles [34] did not show lipid peroxidation and any physiological changes, although activity of catalase, ascorbate and upregulation of heat shock proteins was observed. However, no elevation of lipid peroxidation in rice treated with CeO_2_ nanoparticles (0–500 mg/L) was recorded but ion leakage was observed at higher doses [35].

In recent years, the efficiency of photosystem II (PSII), considered as chlorophyll fluorescence (maximum quantum yield *Fv*/*Fm*), has been used as a diagnostic tool in various studies to check the impact of abiotic stress as well as metal or metal oxide nanoparticle toxicity in various plant species [26, 36–40]. Thus, potential variations in *Fv*/*Fm* act as an indicator of seedling-stock quality/physiological status of plants, in vivo [30, 41–44]. Shaw et al. [40] have shown that CuO nanoparticles reduced shoot and root growth of *Hordeum vulgare* seedlings with passage of time in a dose-dependent manner. However, exposure of CuO nanoparticles in barley seedlings exhibited insignificant alteration in the *Fv*/*Fm* ratio. They have also reported that the CuO nanoparticles induced the release of ROS, membrane damage and overall enzymatic activity not enough to cope with stress at 20-day exposure.

Superparamagnetic iron oxides, Fe_3_O_4_ nanoparticles (SPION) owing to their magnetic properties, are widely used in instruments, medical devices, as drug carrier, and the treatment of many diseases [9]. Like other metal oxide nanoparticles, SPION are cytotoxic to many aquatic organisms and terrestrial plants because they also generate ROS [32]. Bioaccumulation of SPION and other toxic nanoparticles may reach animals through feed and may alter biodiversity. Mushtaq [45] has reported that Fe_3_O_4_ nanoparticles inhibited the seed germination and root elongation of cucumber over a wide range of concentration (500, 2500, 5000 μg/mL). In cucurbits, aggregation of Fe_3_O_4_ nanoparticles occurred followed by their translocation in stem and roots [46].

Replacing biofertilizers by bionanomaterials may sometime be beneficial if they increase the fruit count, seed and biomass without producing toxic effects. Hydroponically grown soybean plants bioaccumulate metal ions, metal nanoparticle such as Zn/ZnO and CeO_2_ [47], which influence soil and microbes associated with plants and biomass [48]. The nitrogen-fixing bacteria are most affected because certain nanoparticles (CeO_2_) eliminate the nitrogen fixation potential and plant growth in soybean. Thus, the soil contaminated with huge quantity of waste material containing a variety of metal/metal oxide nanoparticles may impact both microbes and plants.

Biotransformation of nanomaterials may either enhance toxicity or detoxify the living system [49]. Such transformations are related to redox reaction, sulfidation, phosphorylation and molecular modification [50]. The sulfidation of silver nanoparticles decreased toxicity of *E. coli* owing to lower solubility of Ag_2_S. Similarly, the formation of AgCl from AgNO_3_ in presence of chloride ions also has the same effect.

$$ \begin{array}{ccc}\hfill {\mathrm{AgNO}}_3+\mathrm{HCl}\hfill & \hfill \to \hfill & \hfill \begin{array}{c}\hfill \mathrm{AgCl} + {\mathrm{HNO}}_3\hfill \\ {}\hfill \mathrm{Solid}\hfill \end{array}\hfill \end{array} $$

The plants grown in presence of nanoparticles may absorb and translocate them in different tissues. It has been shown that CuO nanoparticles were reduced to Cu_2_O and Cu_2_S in maize plants [51]. Similar transformation and phytotoxicity of La_2_O_3_ and Yb_2_O_3_ in cucumber have been reported by Ma et al. [52] and Zhang et al. [53]. They were converted to their phosphates in the cucumber roots. The solubility of La_2_O_3_ and Yb_2_O_3_ was enhanced by the organic acids secreted by the cucumber roots. If there are phosphate salts, the biotransformation of oxides to phosphates is enhanced. From a 3-week study of corn plant grown in presence of CeO_2_ nanoparticles, Zhao et al. [34] showed that H_2_O_2_ was accumulated in phloem, xylem and epidermal cells of shoots. Catalase and ascorbate peroxidase activities were also enhanced in the shoot. Since the plants treated with 400 and 800 mg CeO_2_/kg triggered the upregulation of heat shock protein 70 (HSP70) in roots, it is believed that it was due to systemic stress response. The increased activities of enzymes and that of HSP70 are due to the induced reaction against CeO_2_ nanoparticle. CeO_2_ nanoparticle-plant-root interaction and translocation in hydroponically grown wheat and pumpkin plant for 8 days (17–100 nm) at 100 mg/L in the absence and presence of fulvic acid and gum arabic have been reported by Schwabe et al. [54]. The above plants did not exhibit any reduction in root growth. However, the CeO_2_ nanoparticles were translocated in pumpkin shoot but not in wheat plants. SEM and TEM images showed the deposition of nanoparticles on the root surfaces of both the plants which suggested that fulvic acid or gum arabic does not interfere with translocation of CeO_2_ nanoparticles but helps in sticking them to the roots. It has been ascribed to specific alterations in root structure and its interaction with nanoparticles in presence of root exudates [55, 56].

Recently, Rico et al. [57] have shown that CeO_2_ nanoparticles promoted plant development (*H. vulgare*) to the extent of 331% increase in shoot biomass without showing any toxic effect; nevertheless, at higher concentration (500 mg/kg), the plant did not produce grain which is a big loss. Effect of a variety of metal nanoparticles (Ag, Co, Ni) and metal oxide nanoparticles (CeO_2_, Fe_3_O_4_, SnO_2_, TiO_2_) on the translocation of nutrients in tomato plant has been investigated. Higher concentration of metal nanoparticles was found to be accumulated in roots as well as shoots. However, Fe_3_O_4_ nanoparticles promoted root growth and SnO_2_ reduced it [58]. Plant response against some metal oxide nanoparticles is summarized in Table 1.

**Table 1:** Plant response to some metal oxide nanoparticles

Nanoparticle	Size (nm)	Plant	Concentration	Plant response	Key references
CeO_2_	7	Soybean	0, 500, 1000, 2000, 4000 mg/L	Genotoxicity recoded at 2000 and 4000 mg/L concentration; a new band in the roots’ RAPD profile was observed	[47]
	7	Alfalfa, corn, cucumber, tomato	0, 500, 1000, 2000, 4000 mg/L	In corn, tomato and cucumber seed germination was reduced at 2000 mg/L; promoted root elongation for corn and cucumber; reduced root growth of alfalfa and tomato	[60]
	8.0 ± 1.0	Coriander	125 mg/kg	Increased shoot, root length and biomass; increased ascorbate peroxidase activity in roots and catalase activity in shoots	[175]
	<8.0 ± 1.0	Rice	0, 62.50, 125, 250, 500 mg/L	Reduced H_2_O_2_ generation in shoots and roots; increased electrolyte leakage and lipid peroxidation in shoots	[35]
	8 ± 1	Corn	0, 400, 800 mg/kg	No impact on chlorophyll contents and gas exchange	[176]
	8 ± 1	Barley	0, 125, 250, 500 mg/kg	Increased the plant height, chlorophyll contents, biomass, reduced spike production; increased Ca, K, Zn, Mg, Cu, Al, Fe, P and S in grains	[57]
	8 ± 1	Wheat	0, 100, 400 mg/kg	Changes in microstructure of leaf cells, swollen chloroplasts, squeezed nuclei, bent and loosely arranged thylakoids; decreased chlorophyll contents and exhibits variation in protein content	[177]
	10 ± 3.2	*Bacillus thuringiensis* transgenic cotton	0, 100, 500 mg/L	Swollen and destructed chloroplasts, reduced Zn, Mg, Fe and P levels in xylem sap of cotton	[178]
	50–105	Tomato	20 mg/kg	Increased Ca, K, Mg, P in roots; Ca, Mg in stems; decreased Na contents stems; K, Na, P and S in leaves	[58]
	8 ± 1	Wheat	0, 125, 250, 500 mg/L	Changes the amounts S and Mn in grains, amino acid composition and linolenic acid contents	[179]
ZnO	8	Soybean	0, 500, 1000, 2000, 4000 mg/L	No change in germination; genotoxicity recoded at 4000 mg/L concentration; a new band in the roots’ RAPD profile was observed	[47]
	10	Soybean	0–500 mg/kg	Reduced Fe at all treatments; Mg and K were decreased at 500 mg Zn/kg treatment	[180]
	<50	Soybean	500 mg/kg	Reduced roots and shoots; had smaller surface area and volume; no seed formation	[181]
	20	Radish, rape, ryegrass, lettuce, corn, cucumber	2000 mg/L	Reduced root growth and elongation	[19]
	<10	Zucchini	1000 mg/L	Reduced biomass (78–90%)	[182]
	10	Cucumber	400–800 mg/kg	No impact on growth, gas exchange or chlorophyll contents	[183]
	90	Corn	800 mg/kg	Reduced growth and inhibition of arbuscular mycorrhizal fungi	[184]
	10	Alfalfa	250, 500, 750 mg/kg	Reduced root biomass (80%)	[185]
	44.4	Arabidopsis	400, 2000, 4000 mg/L	Reduced seed germination, root elongation and number of leaves	[93]
	<100	Arabidopsis	100 mg/L	Reduced biomass (81.4%), seed germination, 660 up-regulated genes and 826 down-regulated genes	[92]
	<50	Garden pea	100–1000 mg/L	No impact on germination; root length, stem length, leaf surface area, transpiration and root nodulation was affected	[186]
	1.2–6.8	Clusterbean	10 mg/L	Increased biomass (27.1%), shoot length, root length, root area, chlorophyll content and total soluble leaf protein	[98]
	25	Tomato	0–1000 mg/L	Plant height was increased (24%) at 250 mg ZnO/Kg; increased root length in foliar sprayed plants with 250 mg ZnO/L; concentrations above 250 mg ZnO/kg affected root length in both methods of application	[99]
	<100	Wheat	50 mg/kg	Reduced biomass	[103]
	<100	Wheat	500 mg/kg	Reduced root growth, increased reactive oxygen species production	[187]
CuO	<50	Arabidopsis	0, 0.5, 1, 2, 5, 10, 20, 50, 100 mg/L	Reduced biomass, root growth retardation, increased reactive oxygen species production	[116]
	<50	Indian mustard	0, 20, 50, 100, 200, 400, 500 mg/L	Reduced shoot and root growth	[128]
	10–50	Mung bean	0, 20, 50, 100, 200, 500 mg/L	Reduced biomass and root length at all concentrations; reduced chlorophyll content above 100 mg/L; no changes in carotenoid content; increased H_2_O_2_ and lipid peroxidation; increased reactive oxygen species production with increase in concentration; modulations in gene expression	[127]
	<50	Wheat	500 mg/kg	Inhibition in root and shoot growth; produced oxidative stress possibly due to Cu released from nanoparticles, Cu bioaccumulates	[187]
	<50	Squash	0, 100, 500 mg/L	Reduced growth and transpiration (60–70%)	[188]
	<100	Radish, grasses	10, 100, 500, 1000 mg/L	Growth inhibition; DNA damage	[21]
TiO_2_/inorganic bentonite clay	30/1–60	Maize	300, 1000 mg/L	Inhibited hydraulic conductivity, leaf growth and transpiration	[65]
Activated carbon-based TiO_2_	30–50	Tomato	0–500 mg/L	Improved germination, reduced germination time	[189]
	30–50	Mung bean	0–500 mg/L	Improved germination, reduced germination time	[189]
TiO_2_	–	Soybean	0, 0.01, 0.03, 0.05%	Increased height (0.05%) and dry weight	[190]
	<100	Wheat	~91 mg/kg	Reduced biomass, nanoparticles found mostly stick on surface of roots	[103]
	<25	Tobacco	0, 0.1, 1, 2.5, 5%	Reduced biomass, inhibited germination and root length; upregulation of alcohol dehydrogenase and ascorbate peroxidase	[191]
	4–6	Spinach	0.25%	Improved growth; increased glutamate dehydrogenase, glutamine synthetase and glutamic piruvic transaminase activity	[146]
	7–40	Chickpea	2–10 mg/kg	Reduction in electrolyte leakage and malondialdehyde content at 5 mg/kg treatment	[192]
	6.22	*Ulmus elongata*	0.1–0.4%	Increased Cu accumulation in leaves; reduced net photosynthetic rate; increased carbohydrates and lipids	[193]
	27 ± 4	Cucumber	0, 250, 500, 750 mg/kg	Enhanced catalase; activity in leaves; enhanced P and K availability in fruit	[194]
Fe_3_O_4_	20	Pumpkin	500 mg/L	No toxic effect; nanoparticles are translocated throughout the plant tissues, detected in stem and leaves, accumulated on the surface of root	[46]
	7	Cucumber, lettuce	62, 100, 116 mg/L	Low to zero toxicity on germination	[195]
	6	Lettuce, radish, cucumber, spinach, tomato, leek, peppers	0.67 mg/mL	Reduced germination	[196]
	25	Ryegrass, pumpkin	30, 100 and 500 mg/L	Increased root elongation; no uptake; block of aquaporins; oxidative stress	[32]
Fe_2_O_3_	20–100	Sunflower	50, 100 mg/L	No uptake and translocation; reduced root hydraulic conductivity	[158]
	22–67	Arabidopsis	4 mg/kg	Reduced biomass and chlorophyll contents	[197]
	–	Soybean	0, 0.25, 0.5, 0.75, 1.0 g/L	Increased leaf and pod dry weight; increased grain yield (48%)	[167]
	246	Lettuce, radish, cucumber	1000 mg/L	Found to be adsorb on the surface of seed	[198]
Al_2_O_3_	13	Maize, cucumber, carrots, cabbage	2000 mg/L	Reduced root growth	[168]
	–	Corn	2000 mg/L	Reduced root length	[19]
	–	Tobacco	0, 0.1, 0.5, 1%	Increased root length, biomass; decreased leaf count; the seedlings significantly decreased; 1% Al_2_O_3_ exposure has shown extreme increase in microRNA expression	[171]

The main aim of this review article is to present the impact of a number of metal oxide nanoparticles on plants and their distribution in root/shoot. Their toxic effect has also been considered to see if they produced oxidative stress and inhibited the growth of plant, seed or fruit.

#### Cerium Oxide Nanoparticles

The CeO_2_ metal oxide nanoparticles are present in the soil by default due to biosolids disposal from wastewater treatment plant, which are released from the exhaust pipe of automobiles. Priester et al. [59] have studied the impact of nano-CeO_2_ and ZnO nanoparticles on the growth and yield of soybean which is a major crop containing protein. Hydroponically grown soybean plants accumulate metal and metal oxide nanomaterials. It has been found that TiO_2_ and ZnO nanomaterials influence the useful microbes and biomass of the plant especially the nitrifying symbiotic bacteria in the root nodules of soybean and many other plants of fabaceae family. Substantial amount of bioaccumulated ZnO nanoparticles was translocated into leaves and beans while CeO_2_ accumulated into root nodules of soybean plant reduced nitrogen fixation potential and growth. The soil fertility and plant growth are equally affected, and therefore, the controlled amount of metal and metal oxide nanoparticles must only be released in the environment [55]. ZnO and CeO_2_ nanoparticles affect hydroponic plants [18, 47, 60] and microorganisms [7, 48, 61], but their affect on plant growth and crop yield has not been fully explored [62]. There was an increase in the number of pods of soybean treated with cerium oxide (0.1–1.0 g/kg of soil) and nano-ZnO (0.05–0.5 g/kg of soil). It has been noted that CeO_2_ generally reduced the pod and biomass while ZnO nanoparticles increased the pod count and had stimulatory effect on soybean.

Zhao et al. [34] have studied the stress response and tolerance of *Zea mays* to CeO_2_ nanoparticles. It has been reported that CeO_2_ nanoparticles are toxic to bacteria, green algae, fish and soybean plants [47, 63, 64]. The plants and fishes are, therefore, equally affected by the toxicity of these nanoparticles due to contamination of water or soil by CeO_2_. Since the pores of corn primary roots have an average diameter of 6.6 nm, the CeO_2_ nanoparticles with a diameter [65] smaller than these may penetrate root and could be transferred from root to corn shoots [33]. The nanoparticles attached to the roots of corn plant inhibit the water transpiration to the leaves [65]. The above ground parts of plants showed low toxicity. The maize plants were grown for 20 days in soil with CeO_2_ at 400 and 800 mg/kg level, and stress-related responses such as H_2_O_2_, antioxidant enzyme, heat shock protein 70 (HSP70), lipid peroxidation and cell death were recorded every 5 days. About ten times over production and accumulation of H_2_O_2_ in shoots was recorded which indicated a concentration-dependent oxidative stress. In the later stages (after 20 days), the H_2_O_2_ production was reduced, perhaps, the plant growth and adaptation prevented over expression of H_2_O_2_. The enzyme catalase and ascorbate peroxidase detoxify the plant by converting H_2_O_2_ to water and oxygen as shown below:

$$ 2{\mathrm{H}}_2{\mathrm{O}}_2\underset{\mathrm{Ascorbate}\ \mathrm{peroxidase}}{\overset{\mathrm{Catalase}}{\to }}2{\mathrm{H}}_2\mathrm{O}+{\mathrm{O}}_2 $$

The catalase activity in shoots of plants at lower CeO_2_ concentration (400 mg/kg) was 39 times higher but at higher concentration (800 mg/kg), it was reduced to only 30 times. It seems that the enzyme is activated by CeO_2_ but at higher concentration, the activity gradually decreased. Similarly, the ascorbate peroxidase activity also declined with 800 mg/kg dose with a concomitant decrease in H_2_O_2_ level. However, both the enzymes eliminate the excess of H_2_O_2_ to prevent the lipid damage by CeO_2_ nanoparticles.

Biotransformation of CeO_2_ in cucumber has been thoroughly investigated by Zhang and co-workers [66]. During this process of biotransformation, either the toxicity of the nanoparticles is enhanced or it is detoxified [49]. The environmental and the biological systems together alter the toxicity of nanoparticles to organism [67]. Several steps, such as redox reaction sulfidation, phosphorylation and molecular modification, are involved in biotransformation [50]. Biotransformation of Ni(OH)_2_ to Ni^2+^ in plant shoots and leaves was observed by Parsons et al. [68], but no transformation occurred in roots. Some metal nanoparticles or metal oxide nanoparticles are oxidized or reduced depending on the chemical compounds present in certain parts of the plant. For instance, silver nanoparticles were oxidized to Ag(1) by *Lolium multiforum* [69] while CuO nanoparticles were reduced to Cu_2_O and Cu_2_S in maize plants [51]. The toxicity is therefore dependent on the form of element if they are in reduced or oxidized form. Root elongation of cucumber seedlings were inhibited by La_2_O_3_ and Yb_2_O_3_ nanoparticles as a consequence of their biotransformation to rare earth phosphates in the cucumber roots [52, 53] due to the phosphorous compounds exuded by the root of plant.

The CeO_2_ nanoparticles are not toxic to cucumber plants up to 2000 mg/L; however, the major part is accumulated in the root, and about 35% is translocated to leaf and stem, perhaps, because of size constraints. The CeO_2_ nanoparticles mixed with needle-like clusters were found on the outer epidermis of the root. The image of the cluster showed the presence of CePO_4_. It has been reported that the absorbed Ce^3+^ ions on the cell wall of the *Saccharomyces cerevisiae* can react with phosphate released from inside the yeast cells and form Ce(III) phosphate nanocrystallites [70]. Since CePO_4_ is insoluble, it remains on the surface of the root which has been confirmed from TEM/EDS results. The nanocrystals were found to be deposited on the root epidermis and in the intercellular spaces of the cucumber plant. During the biotransformation of CeO_2_ to CePO_4_ in biological system, CeO_2_ undergoes a valence change from Ce^4+^ to Ce^3+^ as a consequence of one electron reduction and formation of CePO_4_.

$$ {}^{4+}{\mathrm{CeO}}_2{\to}^{3+}{\mathrm{CePO}}_4 $$

Both positive and negative results of the CeO_2_ nanoparticles on plant system have been reported [71]. Lopez-Moreno et al. [60] have shown that CeO_2_ nanoparticles of 7 nm were taken up into seedlings of cucumber, alfalfa, tomato and corn at concentrations of up to 4000 mg/L. Particles of 7 to 25 nm were found to be translocated to the shoots of cucumber. It is perhaps the variation in the interaction of nanoparticles with macromolecules present in the root which causes aggregation around the root tips. Besides, uptake and translocation of nanoparticles also depend on the shape, solubility, agglomeration and surface chemistry [5, 22].

Schwabe et al. [54] exposed wheat and pumpkin (hydroponic plant culture) to uncoated CeO_2_ nanoparticle (of 7–100 nm of 100 mg/L) suspension in the presence of fulvic acid and gum arabic. Although the suspension alone was stable, changes in pH, particle agglomeration rate and hydrodynamic diameter in nanoparticles occurred in presence of wheat and pumpkin plants. CeO_2_ nanoparticles were found to be translocated into pumpkin shoots but not in wheat plants. However, no toxic effect was observed in both plants. In a recent experiment, Anderson et al. [72] have chosen ten plant species and found that CeO_2_ and TiO_2_ nanoparticles do not cause widespread acute toxicity during germination and early growth stage. 

Fulvic acid and gum arabic stabilize the CeO_2_ nanoparticles on one hand and reduce their adsorption by root on the other. However, CeO_2_ nanoparticles of 17 to >1 μm are partially available for uptake by pumpkin similar to those found by Zhu et al. [46] and Zhang et al. [73]. Translocation of Ce into shoot of wheat was not detected as the monocots are less likely to take up nanoparticles because water uptake by them (wheat) is only 25% to that of pumpkin.

A recent study on the uptake and accumulation of CeO_2_ nanoparticles in different parts of barley (*Hordeum vulgare* L.) has been made by Rico et al. [57]. The effect of CeO_2_ nanoparticles on the vegetative growth and production of barley grown in soil treated with different quantities of CeO_2_ nanoparticle has been reported (Table 1). It is important to note that at higher dose (500 mg/kg), there occurred rapid shoot development resulting in 331% enhancement of biomass. It is more surprising that at this concentration (500 mg/kg), the barley did not produce grain. It is, however, encouraging that at lower concentration of CeO_2_-amended soil (125 and 250 mg/kg) and also in control-produced grains with large quantity of Ce accumulated in leaves and grains (Table 2) along with P, K, Ca, Mg, S, Fe, Zn, Cu and Al.

**Table 2:** Cerium concentrations (μg/kg dry wt) in different organs of *Hordeum vulgare* cultivated to grain production in cerium oxide nanoparticles-amended soil

Soil treatments (mg/kg)	Leaves	Grains
0-control	571 ± 40	200 ± 5 c
125-*n*CeO_2_-L	595 ± 140	449 ± 51 b
250-*n*CeO_2_-M	524 ± 73	787 ± 58 a
500-*n*CeO_2_-H	701 ± 92	–

Values are means ± SE (*n* = 3). Same letters mean no statistical difference between treatments at Tukey’s test (*p* ≤ 0.05) [57]

Barley treated with CeO_2_ nanoparticles (250 mg/kg) enhanced methionine, aspartic acid, threonine, arginine and linolenic acid contents in the grain. It is clear that the moderate concentration (125–250 mg/kg) of CeO_2_ nanoparticles is highly beneficial while the higher doses (500 mg/kg) are toxic to barley. The accumulation of other metal ions in barley leaves and grains (e.g. P, K, Ca, Mg, S, Fe, Zn) is catalyzed by CeO_2_ nanoparticles. It is perhaps due to the biomolecules coordinated with these metals to make them available as trace elements which also act as nutrient for the plant. CeO_2_ nanoparticles promoted plant growth in soybean and tomato [59, 74] but did not affect the growth of cucumber.

Cerium uptake in root of rice (*Oryza sativa*), wheat (*Triticum aestivum*) and barley (*H. vulgare*) at different doses has been determined. Although CeO_2_ are not translocated in shoots of wheat, they are taken up by rice and barley [35] in fairly large quantity. Recent study [57] suggests that rice, wheat and barley could accumulate CeO_2_ in their root tissues without influencing the rate of germination and root elongation of the seedlings. CeO_2_ nanoparticles may induce modification in plants at molecular levels [35, 75, 76]. Oxidative stress and membrane damage of rice roots have been observed. It has been found from FTIR spectra of the rice, wheat and barley germinated in cerium oxide suspension that changes in amide I and amide II bands (1700–1600 cm^−1^ and 1600–1500 cm^−1^) are significant due to the presence of phenols and proteins [76, 77]. These results suggest that the cerium oxide nanoparticles produce modification in the root xylem of the cereal crops.

The impact of CeO_2_, Fe_3_O_4_, SnO_2_, TiO_2_ and metallic Ag-, Co-, Ni-engineered nanoparticle uptake and translocation in tomato plant has thoroughly been investigated in the recent years [58]. The plant exposed to different quantities of nanoparticles showed different vegetative growth (Table 3). CeO_2_-treated plants showed a very slight increase in stem growth. Fe_3_O_4_ nanoparticles significantly promoted the growth and elongation of tomato plant but reduced its green biomass. SnO_2_ remarkably decreased root growth and the dry stem and leaf weight. Though Fe_3_O_4_ was not significantly translocated into stem and leaf, fairly large amount of iron was found to be deposited in fruit and root of tomato plants. However, titanium, tin and cerium were not translocated even under control or treated plants. It is important to note that in all cases of nanoparticles treated tomato plants, the Ca contents in stem and root increased from 25.6 to 69.8% with respect to control. The bioavailability of nanoparticles depends on the coating, original matter in the soil or even clay because they can alter their behaviour leading to aggregation. Thus, the toxicity of nanoparticles is reduced due to their slow release. It is, therefore, concluded that soil polluted by metals can produce adverse effects if they are present above permissible/tolerance limits.

**Table 3:** Effect of metal and metal oxide nanoparticles on dry matter of roots, stems and leaves of *Lycopersicon esculentum* plants grown in pots

Treatment	Root		Stem		Stem		Root elongation		Plant height	
	g	SD	g	SD	g	SD	cm	SD	cm	SD
Control	1.9 b	0.1	20.5 b	0.9	25.2 a	1.1	22 ab	1.3	98 a	3.8
Ag-NPs	1.6 b	3.3	26.2 a	1.2	24.2 a	0.9	19 b	1.5	82 b	5.3
Co-NPs	1.5 b	0.3	10.3 d	1.1	18.3 b	1.5	15 b	2.8	84 b	4.2
Ni-NPs	1.0 bc	0.3	26.1 a	1.2	12.1 d	0.9	15 b	3.2	93 ab	5.1
CeO_2_-NPs	2.2 ab	0.2	13.1 cd	1.4	15.7 c	0.7	23 ab	2.1	109 a	3.1
Fe_3_O_4_-NPs	4.8 a	0.2	18.1 c	0.8	18.9 b	1.3	25 a	2.3	106 a	3.5
SnO_2_-NPs	0.7 c	0.2	5.4 e	0.7	16.8 c	1.5	11 b	3.7	104 a	3.4
TiO_2_-NPs	1.4 b	0.1	19.2 b	1.1	18.8 b	0.8	17 b	2.1	110 a	4.1

Means followed by a different letter within a row are significantly different at *p* < 0.05 according to Duncan’s multiple range test [58]

Priester et al. [59] have already reported that soybean plants grown in organic farm soil containing ZnO or CeO_2_ nanoparticles absorb and translocate Zn and Ce in all parts of the plant. The results indicated that the low amount of Ce is translocated in soybean pods [78]. The Ce in pods and nodules exists mainly as Ce^4+^ and some amount as Ce^3+^, which suggests that nearly 20% CeO_2_ is reduced to Ce^3+^ [34]. Zhao et al. [34] have shown that Ce was coordinated as CeO_2_ nanoparticles inside the roots of corn plants grown in organic soil amended with alginic acid coated with CeO_2_ nanoparticles. They also showed, from confocal microscopy images, the presence of CeO_2_ nanoparticles in the cell wall of the corn root cortex. They termed it passive uptake of the CeO_2_ nanoparticles. It must be made clear at this juncture that the CeO_2_ nanoparticles are neutral species which cannot coordinate with any ligand carrying lone pair of electrons or negative charges because these electrons are to be partially donated to an electron pair acceptor. However, it is quite likely that the CeO_2_ nanoparticles are translocated to the root cortex with support of the alginic acid coating.

#### Zinc Oxide Nanoparticles

Riesen and Feller [79] have shown the accumulation of zinc in the phloem of wheat plant and also in the soybean grain, but not zinc oxide (ZnO) nanoparticles. Zinc is supposed to be bonded to the oxygen of the carboxyl acid as ZnO [80, 81]. Thangavel et al. [82], from a study of red spruce cell culture, suggested that in living cells, it is more likely that Zn ions bind to the sulphur of phytochelatins rather than the oxygen of organic acids. However, Zn(II) in soybean plants has been found to be associated with oxygen of acids which has also been confirmed on the basis of model compounds [78].

Kopittke and co-workers [83] analyzed cowpea exposed to Zn and found that nearly 65–85% of the zinc was coordinated as a zinc-phytate complex. Phytic acid is present in all beans and is a source of phosphorous storage [84]. It is known that the Zn(II) ions activate the enzyme phytase but it may be bonded to phytic acid to give zinc phytate. Phytic acid is highly unstable and therefore it is stabilized in the form of a metal salt. The free zinc ions are therefore bonded to phytic acid through oxygen giving zinc-phytate complex.

Hu and co-workers [85] have recently investigated the adverse effects of ZnO nanoparticles of 25 nm diameter on the aquatic plant, *Salvinia natans* (L.). During 7-day exposure of plant to different concentrations of ZnO nanoparticles, no significant difference was observed in growth. However, the ZnSO_4_-treated plants showed marked decrease in growth. Generally, 50 mg/L ZnO nanoparticles were found to produce oxidative stress and depressed the photosynthetic pigments. SOD and CAT activities were increased but chlorophyll pigments decreased in the leaves of *S. natans*. It has been already reported that the antioxidant enzymes SOD, CAT and POD can protect plant cells against adverse effects of ROS [26, 28, 29, 32]. While POD acts as scavenger of ROS, SOD and CAT jointly convert O_2_^−^ and H_2_O_2_ to H_2_O and O_2_ and also reduce overall free ·OH radical. Zinc has been reported [86, 87] to increase the biosynthesis of antioxidant enzymes in the duckweed, *Spirodela polyrhiza*. The POD activity was remarkably inhibited by large quantity of ZnO nanoparticles (50 mg/L). In a recent study Zafar et al. [88] have reported that ZnO nanoparticles (500 to 1500 mg/L) negatively affects the *Brassica nigra *seed germination and seedling growth; and also increased antioxidative activities and non-enzymatic antioxidants contents. The toxicity of ZnO nanoparticles depends on the quantum of dissolved zinc in the solution [89, 90]. It is true that only a fraction of dissolved zinc is bioavailable which can be absorbed and translocated in different parts of the plants; nevertheless, the solubility of zinc oxide is pH dependent because being amphoteric in nature, it dissolves in both the acidic and alkaline media as shown below:

$$ \begin{array}{ccc}\hfill \begin{array}{l}\mathrm{ZnO}+2\mathrm{HCl}\hfill \\ {}\mathrm{ZnO}+2\mathrm{NaOH}\hfill \end{array}\hfill & \hfill \begin{array}{c}\hfill \to \hfill \\ {}\hfill \to \hfill \end{array}\hfill & \hfill \begin{array}{l}{\mathrm{ZnCl}}_2+{\mathrm{H}}_2\mathrm{O}\hfill \\ {}{\mathrm{Na}}_2{\mathrm{ZnO}}_2+{\mathrm{H}}_2\mathrm{O}\hfill \end{array}\hfill \end{array} $$

The ZnO nanoparticles diffuse in the plant cells if size is relatively smaller than the pores in the plant cells. Phytotoxicity of ZnO nanoparticles has been investigated by Watson et al. [91] under both acidic and alkaline soils. In acid soil, inhibition of elongation of roots of wheat (*T. aestivum*) was observed whereas phytotoxicity was mitigated in the alkaline soil, although absorption of ZnO nanoparticle was doubled even when Zn concentration in soil was low. Soluble zinc in the acid soil was 200-fold higher and shoot levels were tenfold higher than those in the alkaline soil. Phytotoxicity was observed in soil spiked with humic acid but it did not influence the plant responses. The ZnO nanoparticle aggregation with humic acid provides bioavailable zinc. But these nanoparticles may be distributed to the plant only if they are taken up through diffusion. The plant roots are stunted in the acid soil as the quantity of soluble zinc was 100 times higher in acid soil than the alkaline soil. However, higher dose of Zn (500 mg/L) causes phytotoxicity to the plants.

ZnO nanoparticles (100 mg/L) treated *Arabidopsis* seedlings showed reduced biomass to 81.4 ± 11.5% after 2 weeks [92]. Lee et al. [93] have also reported that ZnO nanoparticle at 400 mg/L inhibited the germination, root growth and leaf development in *Arabidopsis* similar to other plants [20]. ZnO nanoparticles also caused remarkable transcriptomic changes in terms of number of genes and their expression. It has been suggested that ZnO nanoparticles release Zn^2+^ ions and damage root tissues. Under such stress conditions, the plant initiates new root growth as an alternative to the damage by ZnO nanoparticles/Zn^2+^. ZnO nanoparticles promote ROS production in exposed roots [94, 95]. The presence of Zn^2+^ ions is either due to the presence of zinc salt in the ZnO nanoparticle or due to the conversion of ZnO nanoparticles to Zn^2+^ ions. It is, therefore, proposed that stress and defence responses of plants are due to a combined effect of ZnO nanoparticles and Zn^2+^ ions.

Higher toxicity of ZnO nanoparticles with respect to the Zn^2+^ ion in hydroponic solution using *Allium cepa* has been attributed to higher release of ROS [96]. Further, a study with *Vigna unguiculata* in soil amended with either ZnO nanoparticles or Zn^2+^ showed no difference in plant growth, accumulation or speciation between the zinc ion and ZnO nanoparticle treatment [97]. However, foliar exposure of ZnO nanoparticles to *Cyamopsis tetragonoloba* and *Solanum lycopersicum* has revealed a positive response in terms of biomass production, chlorophyll and total soluble leaf protein contents [98, 99].

Effect of citrate-coated Ag and ZnO nanoparticles and uncoated AgNO_3_ and ZnSO_4_ on *Zea mays* L. and *Brassica oleracea* var. *capitata* L. has been explored in vitro. The Ag nanoparticles have been shown to be more toxic to plants than free AgNO_3_. Considerable changes in metaxylem count of maize were observed with Ag nanoparticle, AgNO_3_ and ZnSO_4_ treatments. However, ZnO nanoparticles did not show any significant change in maize. In case of cabbage and maize, the germination and root elongation measurements revealed that nanoparticles were more toxic to plants than the free metal ions [100]. ZnO nanoparticles reduce seed germination [19] and damage tissues [20] in hydroponically grown plants. Kim and co-workers [101] have reported that ZnO nanoparticles at 2000 mg/kg did not affect the root length and biomass production of *Cucumis sativus* grown in a loamy sand soil at pH 5.5. Manzo et al. [102] have reported that ZnO nanoparticles at 286 mg/kg affected the root elongation in *Lepidium sativum* sown in an artificial standard soil. However, Du et al. [103] reported that at only 45.45 mg/kg (5 g/110 kg soil), these nanoparticles reduced the biomass production of wheat (*T. aestivum*) cultivated in loamy clay soil at pH 7.36. X-ray absorption spectroscopic studies have shown absence of ZnO nanoparticles in roots [47, 81]. However, confocal microscopic study showed the presence of FITC-stained ZnO nanoparticles in the stele of corn roots, although these particles were not found in the shoots [33]. It indicated that the ZnO nanoparticles were absorbed and incorporated into the plant transport system.

#### Copper Oxide Nanoparticles

The phytotoxicity of CuO nanoparticles in a 1:1 mixture of CuO and ZnO nanoparticles in plants colonized by *Pseudomonas chlororaphis* in a sand matrix has been investigated by Dimkpa et al. [104]. Bean root growth was inhibited and shoot was elongated by CuO nanoparticles [105, 106]. The CuO nanoparticles were found to release copper to the shoot where maximum copper loading was noted with the minimum dose of 100 mg/kg CuO nanoparticles and lower level of copper with higher doses of 250 and 500 mg/kg. The accumulation of Cu was 10–20-fold higher than the normal level (10 mg/kg). At higher CuO nanoparticle concentration, the major part was accumulated in the root which inhibited its growth. The other essential metal ions in presence of CuO or Cu^2+^ ions are either unavailable to the plant or their absorption was reduced. For instance, accumulation of Fe and Ca in the shoot tissue decreased as a consequence of antagonistic effect of Cu on Fe and Ca [107]. In fact, copper increases the absorption of Fe in animals if iron is available in ferrous form. The reduction of ferric to ferrous occurs by the enzyme ferric reductase but if Cu is in excess, it may be bonded to the enzymes making it unavailable for the reduction of Fe^3+^ to Fe^2+^. As a result, the iron and calcium accumulation in shoot of plant declines [108, 109]. Dimkpa et al. [104] have suggested that Cu(II) is partially reduced to Cu(I) by citrate present in roots of beans and cucumber but chemically Cu(I) is highly susceptible to oxidation in presence of water and air which cannot be avoided in plant system. The redox process is very rapid and hence, the presence of Cu(I) is extremely difficult. CuO/ZnO nanoparticle exposure of bean plants affected both root and shoot. Improved plant growth has been attributed to lower solubility of CuO nanoparticles. It has been suggested that due to alkaline soil, the other metals (Cu and Fe) may be precipitated as their hydroxides and may not be available for absorption by plants. The reduction and accumulation of iron may be due to its hydroxide formation. The exposure of CuO nanoparticles to bean plants reduced Mn, Zn and Ca concentration and increased Na levels in the shoot tissues without disturbing Mn and K levels. In the bacterial culture medium, CuO nanoparticle treatment showed root growth perhaps due to bacteria which formed a protective layer around the root which does not allow copper to be absorbed. However, the plant cells act against the toxic effects of copper nanoparticles and in doing so, certain metals are absorbed and certain others are precipitated. Phytotoxicity of commercial CuO (<50 nm) and Zn nanoparticles (<100 nm) against sand-grown wheat (*T. aestivum*) has been investigated. Since these nanoparticles contained some metallic and non-metallic substances, they may also influence the growth rate of the plant. Changes in shape of ZnO nanoparticles were noted when mixed with sand in aqueous medium.

The sand amended with CuO and ZnO (500 mg/kg) significantly reduced root growth. Dissolved Cu from CuO nanoparticles showed toxic behaviour towards wheat plant but zinc did not influence the shoot growth. CuO and Cu(I)-sulphur complexes were found to be accumulated in the shoot while zinc was detected as Zn phosphate. Oxidative stress in the nanoparticle-treated plants was reflected by an increase in lipid peroxidation and oxidized glutathione and higher peroxidase and catalase activities in roots. The solubility of nanoparticles decreased with increasing aggregation causing morphological changes in ZnO nanoparticles [110]. It has been shown that the amount of Cu and Zn ions released from CuO and ZnO are almost negligible to cause phytotoxicity to plants. Plants grown with nanoparticles showed increased accumulation of Cu and Zn (20 fold Cu and 24 fold Zn) which altered root metabolism in wheat plants. Both CuO and ZnO nanoparticles have been detected in shoot of the plants. However, the quantitative difference between the two metals is mainly due to their solubility/diffusion. The zinc as zinc-phytate accumulates in the plants.

CuO nanoparticles have been shown to induce DNA damage in plants [21]. Growth inhibition in radish (*Raphanus sativus*), perennial ryegrass (*Lolium perenne*) and annual ryegrass (*Lolium rigidum*) under laboratory conditions has been reported. Germination of radish seeds in presence of CuO nanoparticles induces substantial accumulation of mutagenic DNA lesions. Radish and similar other plants produce oxygen-derived species (O_2_^−^, H_2_O_2_,·OH) during germination [111]. H_2_O_2_ enhances seed germination but in presence of peroxidase or transition metal ions such as iron or copper produce an excess of OH via the Fenton reaction [112]. It is therefore suggested that copper ions produced from CuO nanoparticles may catalyze the formation of OH. CuO nanoparticles inhibited the radish root growth to the extent of 79% which is relatively much larger than that observed for Cu^2+^ ions alone. The stunted growth has been observed mainly in the root/shoot [113].

CuO nanoparticles have been shown to be cytotoxic and genotoxic [114, 115] to mammalian cells. It is thought to be due to nanoparticles which produce oxidative stress within living cells and cause DNA damage in plants or animals. Nair and Chung [116] have studied the impact of CuO nanoparticles on the growth of *Arabidopsis thaliana* and changes at molecular level. The seedlings were exposed to different concentrations of CuO nanoparticles (0.5 to 100 mg/L) for 3 weeks under laboratory conditions. Total chlorophyll contents were significantly reduced at all concentrations starting from 2 to 100 mg/L. Root growth was reduced even with 0.5 mg/L CuO nanoparticles. Superoxide and hydrogen peroxide increased in roots and leaves with increasing concentration of nanoparticles in plant. Oxidative stress, sulphur assimilation of glutathione and proline biosynthesis were also influenced by CuO nanoparticle exposure. In another study, *A. thaliana* plants exposed to cerium oxide and indium oxide showed reduction in plant biomass and total chlorophyll contents [117]. The increase in anthocyanin (the flavonoid) concentration in *A. thaliana* plant exposed to CuO nanoparticles may be due to oxidative stress. Anthocyanin acts as antioxidant to protect the plant cells against ROS-induced oxidative stress [29]. It is obvious that when a foreign matter is absorbed by the plant, it acts against this material through defensive mechanism for protection. Thus, it produces antioxidants which act as scavenger of ROS. As a result of stress by CuO nanoparticles, lignin was also deposited in *A. thaliana*.

It has been proposed that CuO nanoparticles would have been translocated via the vascular tissues and subsequently dissolved to produce Cu ions which resulted in deposition of lignin. Translocation of CuO nanoparticles is apparent but the production of Cu ions by dissolution is impossible because generation of Cu ions from copper nanoparticles is a redox process which requires a reducing agent such as hydrogen, phenol, protein or an acid. However, CuO being weakly basic dissolves in HCl to give ions as follows:

$$ \begin{array}{lll}\begin{array}{l}\mathrm{CuO}+2\mathrm{HCl}\hfill \\ {}{\mathrm{Cu}\mathrm{Cl}}_2\hfill \end{array}\hfill & \begin{array}{c}\hfill \to \hfill \\ {}\hfill \to \hfill \end{array}\hfill & \begin{array}{l}{\mathrm{Cu}\mathrm{Cl}}_2+{\mathrm{H}}_2\mathrm{O}\hfill \\ {}{\mathrm{Cu}}^{2+}+2{\mathrm{Cl}}^{-}\hfill \end{array}\hfill \end{array} $$

**Fig. 1:**
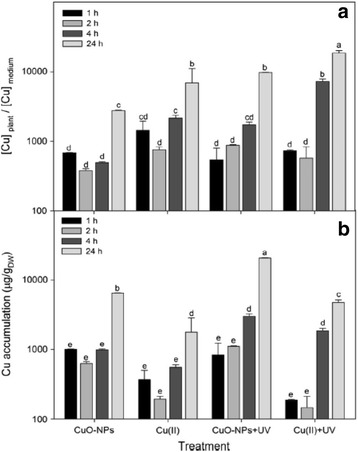
**a** Accumulation ratios ([Cu]_plant_/[Cu]_medium_) and **b** accumulation of Cu in shoots of *Elodea nuttallii* exposed to 256 μg/L Cu(II) or 10 mg/L CuO nanoparticles for up to 24 h. UV was applied additionally to test for effects on Cu accumulation. UV exposure lasted maximal 8 h: in the 24-h treatment, a 16-h period without UV followed the 8-h UV treatment before sampling. Different *letters* indicate statistically significant differences between the values as obtained by ANOVA and Tukey’s post hoc test (*p* < 0.05), where the letter *a* is assigned to the groups with the highest mean values [118]

A comprehensive study of uptake and toxic effects of CuO nanoparticles, Cu^2+^ ions and also in combination with UV radiation has been done on the aquatic macrophyte, *Elodea nuttallii* [118]. Growth of the plants was inhibited when treated with CuSO_4_ or CuO nanoparticles. However, the amount of copper accumulated in *E. nuttallii* was lower in CuSO_4_-treated plants than those treated with CuO nanoparticles (Fig. 1). The difference has been attributed to the solubility of Cu^2+^ in CuO nanoparticle medium. Surprisingly, the relation between accumulated Cu and dissolved Cu^2+^ was higher in plants exposed to 256 μg/L Cu^2+^ than those exposed to 10 mg/L CuO nanoparticles containing nearly 2.0 mg/L dissolved Cu (Fig. 1). The accumulated Cu is directly proportional to the amount of Cu dissolved in the medium but it is not related to higher concentration of Cu in the solution [119]. Perhaps at higher concentration, agglomeration occurs which is prevented from absorption. When the dissolved copper is in small quantity, it has greater degree of freedom for movement and can be accumulated in different parts of the plant. However, enhanced Cu uptake in plants exposed to CuO nanoparticles with dissolved Cu concentration has also been reported which is contradictory to above results [120]. It has been suggested that plants exude some acid to dissolve additional Cu from CuO nanoparticles which is transported and accumulated in different parts of the plant [121]. Formation of large agglomerates prevents the dissolution of Cu. It is interesting that Cu accumulation is enhanced, under UV radiation, in shoots after 4 h but there is no direct evidence of enhanced solubility of Cu^2+^ in CuO nanoparticle suspension. Accumulation of Cd under UV radiation has been ascribed to membrane damage of the plant [122]. Rai et al. [123] suggested altered membrane permeability due to lipid peroxidation in cell membranes of UV-exposed cells in cynobacteria. In plants, under UV radiation, photosynthetic capacity is strongly reduced. When higher quantity of Cu is accumulated in plants, the response of the oxidative stress-related enzymes peroxidase and superoxide dismutase is also high.

Duckweed exposed to CuO nanoparticles showed inhibition of photosynthetic activity due to the Cu^2+^ ions released from it [120]. Andreotti et al. [124] have found that *Phragmites australis* can relocate Cu from roots to shoots. CuO nanoparticle exposure to cotton and Bt cotton has shown significant alterations of the concentrations of indole-3-acetic acid and abscisic acid [125]. Cu-based nanoparticles have been found to increase P and S in alfalfa shoots but decreased Fe and P in lettuce shoots [126]. Carotenoids remained unchanged and chlorophyll reduction began at 100 mg/L CuO nanoparticles in mung beans [127]. Carotenoid contents diminished at 400 mg/L CuO nanoparticles in soybean plants, but chlorophyll started decreasing at 400 mg/L [116]. In another study, CuO nanoparticles reduced carotenoids and chlorophylls in mustard [128] while Cu-based nanoparticles did not affect chlorophyll production in cilantro [129].

#### Titanium Oxide Nanoparticles

Although TiO_2_ is used in many consumable materials like cosmetics, sunscreen and colouring matter in medicine, paints, surface coating and water contamination process, it causes pulmonary inflammation in man if inhaled [130–132]. The quantity and exposure time of TiO_2_ significantly increase toxicity. Clément and co-workers [133] have reported that TiO_2_ nanoparticles (25 nm) with anatase crystal structure are more toxic to cladocerans, algae, rotifers and plants. At high concentration, they promoted growth of roots in plants. Since the rutile crystalline structures of TiO_2_ nanoparticles form aggregates in aqueous medium and are less available for absorption, they are less toxic than the anatase TiO_2_ (1 μm). When the exposure time is increased from 24 to 72 h, toxic effect is enhanced. However, at higher concentration (100 mg/L) of anatase TiO_2_ nanoparticles, the germination of seeds and root growth of flax seeds was enhanced. The positive effect has been suggested to be due to antimicrobial properties of anatase TiO_2_ which increases plant resistance to stress. Similar results have also been obtained by Zheng et al. [134].

Larue et al. [135] have studied the effect of anatase and rutile TiO_2_ on the development and germination of wheat (*Triticum aestivum*) over a period of 7 days. It has been observed that anatase TiO_2_ nanoparticle of diameter lower than 140 nm is accumulated in wheat roots. They are translocated to leaves if their diameter is smaller than 36 nm even if it is below the detection limit. TiO_2_ nanoparticles neither dissolve nor their crystal phase undergoes any change during translocation. The exposure of wheat plant to 14 and 22 nm TiO_2_ nanoparticles causes enhancement in root elongation without influencing seed germination, vegetative growth, photosynthesis or redox reaction. These are short-term effects of TiO_2_ nanoparticles but during the whole cycle of the plant, it may have some adverse effects.

Accumulation and translocation of TiO_2_ nanoparticles in plants suggest that it is not biodegradable. Long-term deposition may interfere with biological function of plants. Servin and co-workers [136] have studied the effect of TiO_2_ nanoparticles in cucumber plants (*Cucumis sativus*) over a large accumulation range (0–4000 mg/L). They found that root was significantly increased up to a concentration of 500 mg/L but above this concentration, it ceases to grow further. Nitrogen in the root was converted to organic nitrogen showing an increase of about 51.1%, compared to control. It is thought that TiO_2_ nanoparticles promote plant root growth by stimulating nitrogen accumulation.

Nanomaterials of the same metal with different structural motifs have different effects on plants, although there is no distinction in their chemical behaviour. Of the three crystalline structures of TiO_2_ nanoparticles (anatase, rutile and brookite), anatase exhibits the highest catalytic activity [137] and can inhibit the growth of many microbes such as algae, fungi and bacteria. It promotes carotene and chlorophyll synthesis in cucumber. TiO_2_ nanoparticles increase the Hill reaction and chloroplast activity by enhancing light absorption in chlorophyll a, electron transfer and oxygen evolution rate in spinach leaves [138–142]. Ba [143] showed that nano-TiO_2_ solution can inhibit the germination and growth of cucumber seedlings due to accumulation of nanoparticles. However, rutile TiO_2_ nanoparticles can protect chloroplast membrane against reactive oxygen and free radicals and enhance the protective activities of antioxidant enzymes such as SOD, CAT and POD [144, 145].

Nano-anatase TiO_2_ promotes the activity of spinach nitrate reductase and accelerates the conversion of nitrogen as nitrates or ammonium salts to organic nitrogen (protein, etc.) [146] but the uptake of other essential metals like Mg and Mn is not affected. TiO_2_ nanoparticles, however, stimulated the synthesis of carbohydrates and lipid as a consequence of stress caused by nanomaterials. Tomato seeds treated with TiO_2_ nanoparticles and Ag nanoparticles did not influence the germination, perhaps, the thick seed coat did not allow the absorption of nanomaterials. The Ag nanoparticles, at higher concentration (500 to 5000 mg/kg), are toxic to tomato plant during germination and the plant could not grow to full length. Silver nanoparticles in the mature tomato plants showed lower chlorophyll contents, higher SOD activity and less fruit productivity, while nanoTiO_2_ exhibited higher SOD activity at the highest concentration (5000 mg/kg). Both nano-Ag and nano-TiO_2_ were also taken up into plant stem, leaves and fruits [147]. The soil fortified with TiO_2_ nanoparticles enhances the chlorophyll content, POD, CAT and nitrate reductase of many plant species [148, 149].

#### Iron Oxide Nanoparticles

Hazeem et al. [150] have studied the effect of Fe_3_O_4_ nanoparticles on the growth of *Picochlorum* sp. in aqueous medium. The small (20 and 40 nm) and large (>100 nm) particles at 200 mg/L were used to examine the growth and chlorophyll content at different stages of growth of algae. The nanoparticles of 20 nm with different concentrations promoted the algal growth besides their aggregation and sedimentation. It prompted the authors to believe that this phenomenon can be used in bioremediation of environment from nanoparticles.

The metal oxide nanoparticles are more toxic to microorganisms than the bulk material of the same metal [151], despite the fact that bulk Fe_3_O_4_ is used as an algal fertilizer and also a source of iron as nutrient [152]. The smaller nanoparticles penetrate into plant cells or microbial cells but larger ones adhere to cell wall causing agglomeration.

The toxicity of SPION against an aquatic plant *Lemna gibba* has been investigated [153]. It was found that chlorophyll contents decreased, photosynthetic activity reduced and growth was hampered. The toxicity of nanoparticles is mainly dependent on their size and solubility in aqueous medium. Inhibitory effects of Fe_3_O_4_ nanoparticles after 6 days on cucumber (*Cucumis sativus*) seed germination and root elongation have also been reported [45]. Germination index of seeds was decreased by exposure of Fe_3_O_4_ nanoparticles at 500, 2500 and 5000 μg/mL. The effect of manufactured iron oxide nanoparticles on uptake and accumulation in pumpkin (*Cucurbita maxima*) plants grown hydroponically was investigated by Zhu and co-workers [46]. They showed that different amounts of Fe_3_O_4_ nanoparticles were taken up and translocated throughout the root, stem and leaves and indicated the nanoparticle transport pathways and bioaccumulation into the plant system. Chen et al. [154] have shown that Fe_3_O_4_ nanoparticles caused a decrease in net photosynthetic rate and chlorophyll a content when alga *Chlorella vulgaris* was exposed for 72 to 100 h and 200 × 10^3^ μg/L of Fe_3_O_4_ nanoparticles. Iron oxide nanoparticles at 3.2 mg/kg showed reduced mycorrhizal clover biomass by 34% by reducing the glomalin content and root nutrient acquisition of *Arbuscular mycorrhizal* fungi [155]. Wang et al. [32] reported that Fe_3_O_4_ nanoparticles induced oxidative stress as compared to Fe_3_O_4_ bulk particles in the ryegrass and pumpkin roots and shoots as indicated by increased SOD and CAT activities and lipid peroxidation. Authors have shown that the tested Fe_3_O_4_ nanoparticles were unable to translocate in the ryegrass and pumpkin plants. The clogging effects of iron oxide nanoparticles reduce the root hydraulic conductivity by inhibiting the adequate water uptake [117, 156, 157]. Iron oxide nanoparticles have been found to reduce macronutrients such as Ca, K, Mg and S in sunflower’s shoots [158]. It has been suggested that it was due to the water blocking effects of nanoparticles, which altered the dissolved nutrients in water. Wang et al. [32, 159] have reported an increase in lipid peroxidation and attributed to Fe_3_O_4_ blockage of the aquaporins and disturbance of the respiration rate in the root. Many other studies on the use of iron oxide nanoparticles have shown reduced accumulation of chlorophylls in the leaves. However, this response is also associated with the reduction of the root hydraulic conductivity and the transport of dissolved nutrients, particularly of Mg since this nutrient is an essential constituent of the chlorophyll pigment [158, 117, 156, 160].

**Fig. 2:**
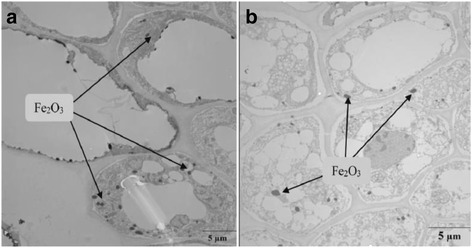
Transmission electron microscopy images of root sections of non-transgenic cotton (**a**) and Bt transgenic cotton (**b**) plants after 10 days of treatment with Fe_2_O_3_ nanoparticles [162]

In a recent study, Shankramma et al. [161] have shown enhanced growth parameters of *S. lycopersicum* exposed to Fe_2_O_3_ nanoparticles. They found nanoparticles deposited preferentially in root hairs, root tips followed by nodal and middle zone of plant. They have suggested biomineralization of nanoparticles due to rich phytochemicals in plants. However, in another study, Nhan et al. [162] showed that 1000 mg/L Fe_2_O_3_ exposure to Bt transgenic and non-transgenic cotton exhibited presence of dark dots (particles) primarily localized in the endodermis and vascular cylinder (Fig. 2). Absorption of Fe_2_O_3_ nanoparticles and their aggregation in the roots were apparent. Iron contents in the shoots and roots increased with increasing concentration of Fe_2_O_3_ nanoparticles. It has been suggested that the bioaccumulation of Fe_2_O_3_ nanoparticles in Bt transgenic and non-transgenic cotton may cause potential risk for environment and human health. Affect of citrate-coated Fe_3_O_4_ nanoparticles on hydroponically grown wheat plant (*T. aestivum*) has recently been studied by Iannone et al. [163]. TEM images of root section showed the deposition of Fe_3_O_4_ in epidermal cell wall via apoplastic route. Fairly large quantities of magnetite (2.01–8.07 mg/g) iron were found in wheat roots. Since no paramagnetic signal was detected in stem and leaves, it suggested that nanoparticles were not translocated through vascular tissues. However, Fe_3_O_4_ nanoparticles affect the germination, chlorophyll content and plant growth and also did not produce lipid peroxidation or H_2_O_2_ accumulation. Antioxidant enzyme activity of plant reasonably increased in root and shoot which indicates that the Fe_3_O_4_ nanoparticles are not toxic to wheat plant under the given experimental conditions. The efficacy of Fe_2_O_3_ nanoparticles as iron fertilizer for peanut (*Arachis hypogaea*) has been studied to check if it can replace the conventional iron fertilizer [164]. The Fe_2_O_3_ nanoparticles and Fe_2_O_3_-EDTA were found to increase root length, height and biomass of the plant by regulating phytohormones and antioxidant enzyme activity. Fe_2_O_3_ nanoparticles are adsorbed onto the soil increasing easy availability of iron to peanut plant. The adsorption of nanoparticles in presence of organic matter is enhanced. It has been demonstrated from hydroponically grown spinach in presence of Fe_2_O_3_ nanoparticle and Fe(NO_3_)_3_·9H_2_O salt that the growth rate of plant is dose and time dependent [165]. The lengths of spinach stem under different concentrations of Fe_2_O_3_ (100, 150, 200 mg) were 1.45, 1.91 and 2.27-fold greater than those of the control after 45 days. There was, however, no significant change in the plant growth treated with Fe(NO_3_)_3_. The vegetative growth in the non-fruit-producing plants/vegetables such as cabbage, radish and beat root is highly useful because they increase the rate of photosynthesis. Hu et al. [166] found uptake of Fe_2_O_3_ nanoparticles in plant roots but no translocation from roots to shoots was observed. In the case of soybean, an increase in leaf and pod production due to uptake of Fe_2_O_3_ has also been shown in previous studies [167]. The mechanism of uptake of Fe_2_O_3_ nanoparticles has been explained in terms of reduction of Fe_2_O_3_ to Fe^2+^. Since Fe^3+^ is insoluble in aqueous medium, it is converted to Fe^2+^ in the soil when it became slightly acidic and absorbed. In hydroponic condition, the acidity is produced due to the addition of the nutrient, NH_4_H_2_PO_4_. The presence of iron phosphate has been evidenced from IR spectrum of the nutrient (containing NH_4_H_2_PO_4_ and Fe_2_O_3_) which is absorbed and translocated to different parts of the plant.

#### Aluminium Oxide Nanoparticles

In the beginning, Yang and Watts [168] reported inhibition of root elongation in maize, cucumber, soybean, cabbage and carrot exposed to 13 nm Al_2_O_3_ nanoparticles. Thereafter, Lin and Xing [19] observed no phytotoxicity after 60-nm-sized Al_2_O_3_ nanoparticle application in radish, rape, ryegrass, lettuce and cucumber, although root elongation was reduced by 35% in maize. In contrast, studies with *Phaseolus vulgaris* and *Lolium perenne* have shown that 100-nm-sized Al_2_O_3_ nanoparticles had no adverse effect on plant growth [169]. Phytotoxicity of 150-nm-sized Al_2_O_3_ nanoparticles in *A. thaliana* has been investigated by Lee et al. [93] and no toxic effect was observed. Sadiq et al. [170] have reported the negative effect of below 50-nm-sized Al_2_O_3_ nanoparticles on the development of microalgae (*Scenedesmus* sp. and *Chlorella* sp.). Effect of Al_2_O_3_ on the growth and development of *Nicotiana tabacum* and role of microRNA has been investigated [171]. It has been observed that as the concentration of Al_2_O_3_ nanoparticles increases from 0.1 to 1.0%, the root length, average biomass and the leaf count of each tobacco seedlings decrease. The seedlings form multiple roots with increasing concentration of Al_2_O_3_, perhaps, as defensive mechanism to avoid contact with excess nanoparticles. They proposed that the microRNA genes were upregulated and played a key role in plants’ ability to withstand under Al_2_O_3_ stress. Al_2_O_3_ nanoparticles (40 nm) had no effect on root elongation of *Triticum aestivum* [172]. However, in a recent study, Yanık and Vardar [173] have reported that Al_2_O_3_ nanoparticles inhibit root elongation, callose formation, lignin deposition, cellular deformation, enhancement of peroxidase activity, decrease in total protein content and DNA fragmentation in *T. aestivum*. It has been suggested that the negative effects of Al_2_O_3_ nanoparticles were time and dose dependent. Impact of Al_2_O_3_ nanoparticles of 30–60 nm on soybean plant under flooding condition has been investigated by Mustafa and Komatsu [174]. The root length was found to increase while proteins related to glycolysis were suppressed. Al_2_O_3_ nanoparticles mediated the scavenging activity of cells by regulating the ascorbate/glutathione pathway. The results suggested that Al_2_O_3_ of varying size and shape affects mitochondrial proteins. Since it is a very short-term experiment on soybean seedlings, the plants may recover and tolerate the adverse effects of Al_2_O_3_ when they are fully grown.

## Conclusions

The inadvertent use and release of nanomaterials into the environment affect plant growth and developmental process from seed germination to crop/fruit production. A variety of metal oxide nanoparticles (CeO_2_, ZnO, CuO, TiO_2_, Fe_3_O_4_ and Al_2_O_3_, etc.) has been examined against seed germination, growth of shoot/root, biomass production and physiological and biochemical activities. They have shown beneficial as well as adverse effects on the plant system and production. Plants absorb them on the surface and subsequently translocated and stored in different tissues. Quite often, the innocuous types of nanoparticles at low concentration have not exhibited any significant adverse effect and seem to be beneficial. However, at higher concentration, it produces stress/toxicity and enhances the generation of reactive oxygen species which results in the disruption of the cellular metabolism. In those conditions, plants protect cellular and sub-cellular system from the cytotoxic effects of active oxygen radicals with antioxidative enzymes and non-enzymatic components. Most of the studies have been carried out on the early developmental stages of the plant which requires full-term study to reach final results. It is hoped that some of the nanomaterials will minimize the use of toxic chemicals and fertilizers in near future. Additionally, detailed research investigations are required to examine the impact of these nanoparticles on the environment and human health.
